# Serial cerebrospinal fluid concentrations of high mobility group box 1 in bacterial meningitis: a retrospective cohort study

**DOI:** 10.1186/s12879-025-10476-7

**Published:** 2025-01-23

**Authors:** Takeshi Matsushige, Hirofumi Inoue, Madoka Hoshide, Fumitaka Kohno, Hikaru Kobayashi, Kiyoshi Ichihara, Takashi Ichiyama, Shunji Hasegawa

**Affiliations:** 1 Department of Pediatrics, Yamaguchi University Graduate School of Medicine, 1-1-1 Minamikogushi, Ube, Yamaguchi 755-8505 Japan; 2 Department of Clinical Laboratory Sciences, Faculty of Health Sciences, Yamaguchi University Graduate School of Medicine, 1-1-1 Minamikogushi, Ube, Yamaguchi 755-8505 Japan; 3 Division of Pediatrics, Tsudumigaura Medical Center for Children With Disabilities, 752-4 Kume, Shunan, Yamaguchi 745-0801 Japan

**Keywords:** Bacterial meningitis, Cerebrospinal fluid, High mobility group box 1, Cytokine, Prognosis

## Abstract

**Background:**

Bacterial meningitis (BM) is a life-threatening central nervous system infection with potential for severe neurological sequelae. High mobility group box 1 (HMGB1) is known as a late inflammatory mediator associated with lethal pathology. This study aims to investigate the serial cerebrospinal fluid (CSF) concentrations of HMGB1 in children with BM and its relationship to neurological prognosis.

**Methods:**

This retrospective cohort study included children with BM, aseptic meningitis (AM), and controls. CSF samples were collected serially from patients with BM and once from those with AM and controls. HMGB1 and interleukin-6 (IL-6) concentrations were measured using ELISA and bead-based multiplex assays, respectively. Statistical analyses included Mann–Whitney U tests, Kruskal–Wallis tests, and three-way ANOVA to evaluate differences among groups and over time.

**Results:**

HMGB1 levels in the CSF of children with BM were significantly higher than in those with AM and controls (p < 0.001). Inflammatory cytokine IL-6 levels decreased after treatment; however, HMGB1 levels remained elevated in half of the BM patients. Notably, a patient with neurological sequelae exhibited a delayed elevation of HMGB1 until the latest time points. Three-way ANOVA revealed significant differences in the time course of IL-6 and HMGB1 among individuals (p = 0.018).

**Conclusions:**

Elevated CSF HMGB1 levels persist in some children with BM even after treatment, particularly in those with poor neurological outcomes. These findings suggest that delayed elevation of HMGB1 may contribute to severe inflammation and poor prognosis in BM. Further research into HMGB1 as a potential therapeutic target in BM is warranted.

**Supplementary Information:**

The online version contains supplementary material available at 10.1186/s12879-025-10476-7.

## Introduction

Bacterial meningitis (BM) is a serious and life-threatening infectious disease of central nervous system. Symptoms of BM include fever, convulsion, altered consciousness, headache, and vomiting. It is indispensable to diagnose and start the empiric antibiotic therapy as soon as possible. It has severe neurological sequelae in 12 to 17% of survivors, and milder impairment of neurological function occurs in 20 to 32% [1–3]. BM is primarily caused by bacterial invasion to central nervous system through the blood brain barrier. High levels of inflammatory response, so called cytokine storm, play an important role in the pathogenesis of BM, and adjunctive treatment of dexamethasone improves neurological outcomes [4]. However, its use is not universally accepted as a standard of care, as its effects on mortality remain uncertain [4].

High mobility group box 1 (HMGB1) is a main component of non-histone DNA-binding protein and is widely distributed in all mammalian tissues [5]. Previously, HMGB1 was known to be involved in maintaining nucleosome structure and regulation of gene transcription [6, 7]. Recently, HMGB1 has been identified as a late-phase inflammatory mediator in sepsis, persisting during prolonged inflammation and contributing to poor outcomes [8–12], while cytokines such as IL-6 act as early-phase mediators and play a key role in the cytokine storm associated with BM. Elevated serum HMGB1 concentrations have been reported in septic shock, acute lung injury, disseminated intravascular coagulation, and surgical operation [10, 13]. Notably, HMGB1 levels were found to increase after IL-6 levels in sepsis [11], and anti-HMGB1 antibody have been shown to reduce mortality, suggesting HMGB1 as a potential therapeutic target [14–16].

We determined cerebrospinal fluid (CSF) HMGB1 concentrations and kinetics to evaluate the pathogenesis of BM and compared CSF HMGB1 and IL-6 levels among BM, aseptic meningitis (AM), and controls. By analyzing both IL-6 and HMGB1 serially, we aimed to better understand their temporal relationship and potential contributions to disease progression and neurological outcomes in BM.

## Materials and methods

### Patients

Informed consent was obtained from the parents of the patients and controls enrolled in this study. This study was approved by the Institutional Review Board at Yamaguchi University Hospital (H26-9).

**Fig. 1:**
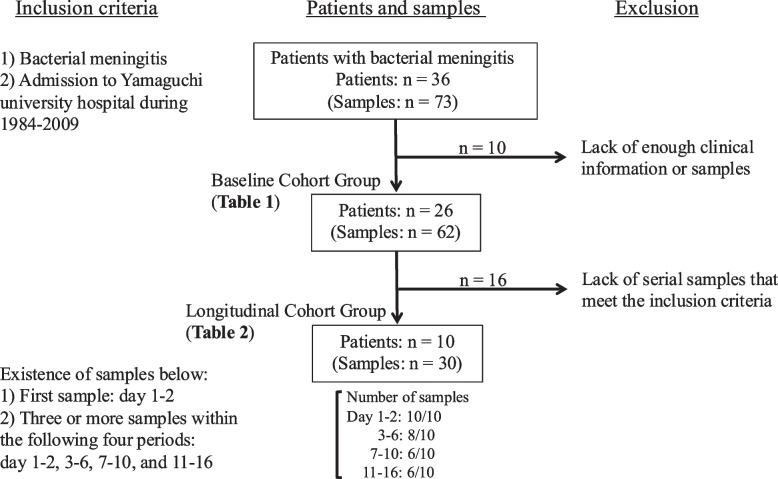
Flowchart of the selection of patients with bacterial meningitis (BM)

This study included two analysis groups: the Baseline Cohort Group and the Longitudinal Cohort Group, with the Longitudinal Cohort Group representing a subset of the Baseline Cohort Group (Fig. 1). Each group was defined by specific inclusion and exclusion criteria to address different study objectives.

#### Baseline Cohort Group in BM (Fig. 1, Table 1)

**Table 1:** Demographics, causative agents, and prognosis of bacterial meningitis in the baseline cohort group, aseptic meningitis, and control

	Bacterial meningitis	Aseptic meningitis	Control	*P* value
Number of patients	26	13	12	
Number of samples	62	13	12	
Years of age, median (range)	0.8 (0.1–3.5)	3.4 (0.1–7.0)	1.4 (0.1–5.1)	†
Sex, male: female	14: 12	6: 7	6: 6	0.89
Sampling day from onset [day]	1–40	1		
Causative agents	*Haemophilus influenzae* (12)	*Enterovirus* (3)		
	*Streptococcus pneumoniae* (6) *Escherichia coli* (4) Others (4)	*Mumps virus* (1) *Mycoplasma pneumoniae* (1) Unknown (8)		
Prognosis	No sequelae (20)	No sequelae (13)		
	Death (1)			
	Hearing impairment (3)			
	Intellectual disability (3)			
	Epilepsy (2)			
	Hydrocephalus (1)			

a significant Kruskal–Wallis test result for three-group comparison). There were no significant differences between the others

^†^*p* = 0.0018, bacterial meningitis vs. aseptic meningitis (post-hoc comparison using Bonferroni-corrected Mann–Whitney U test following

##### Objective

To compare HMGB1 and cytokine levels among BM, AM, and control groups, and within the BM group by bacterial species, neurological outcomes, and correlations between biomarkers.

Inclusion criteria:

1. Diagnosis of BM.
2. Admission to Yamaguchi University Hospital between 1984 and 2009.

##### Exclusion criteria

Insufficient clinical information or incomplete samples.

##### Cohort description

Initially, 36 children aged 0–18 years with BM were identified. After excluding 10 patients due to insufficient clinical information or incomplete samples, 26 patients (median age 0.8 years [interquartile range (IQR): 0.3–1.2], range 0.1–3.5 years) were included for analysis. CSF cultures from 26 patients with BM yielded *Haemophilus influenzae* (*H. influenzae*, *n* = 12), *Streptococcus pneumoniae* (*S. pneumoniae*, n = 6), *Escherichia coli* (*E. coli*, *n* = 4), Group B *Streptococcus* (GBS, *n* = 1), *Listeria monocytogenes* (*n* = 1), *Proteus mirabilis* (*P. mirabilis*, *n* = 1), and *Methicillin-Resistant Staphylococcus epidermidis* (*n* = 1). The patients were treated with empiric antibiotics in adequate doses according to ages and basic diseases. Adjunctive dexamethasone therapy was administered according to national clinical guidelines in Japan (0.6 mg/kg/day in four intravenous doses for two or four days, started before antibiotics) [17]. One patient died and 5 patients had neurological sequelae (2 epilepsy, 3 intellectual disability, 3 sensorineural hearing impairment, and 1 hydrocephalus, which were overlapped).

#### Longitudinal cohort group in BM (Fig. 1, Table 2)

**Table 2:** Clinical features of the representative patients with bacterial meningitis in the longitudinal cohort group

Patient No	Age	Causative agents	Blood			Cerebrospinal fluid			Treatment		Prognosis
			WBC [10^6^/L] on admission	CRP [mg/dL] on admission	max CRP [mg/dL]	Cell [/μL]	Protein [mg/dL]	Glucose [mg/dL]	Antibiotics	Dexamethasone	
1	9–10 months	*H. influenzae*	10,700	20.3	20.3	1,025	175	13	CTX, MEPM	0.6 mg/kg, 2 days	No sequelae
2	9–10 months	*H. influenzae*	3,700	7.9	30.5	13,184	484	10	CTRX, PAPM/BP	0.6 mg/kg, 2 days	No sequelae
3	1–2 years	*H. influenzae*	16,280	22.2	31.5	2,800	74	41	CTX, MEPM	0.6 mg/kg, 2 days	No sequelae
4	5–6 months	*H. influenzae*	8,700	24.1	24.1	403	50	30	CTX, MEPM	0.6 mg/kg, 2 days	No sequelae
5	3–4 years	*H. influenzae*	9,560	20.2	20.2	11,600	432	1	CTX, MEPM	0.6 mg/kg, 2 days	No sequelae
6	5–6 months	*S. pneumoniae*	10,090	15.7	15.7	1,552	238	9	CPR, PAPM/BP	0.6 mg/kg, 2 days	Hearing impairment
7	1–2 years	*S. pneumoniae*	6,590	34.1	34.1	512	118	2	CTRX, PAPM/BP	0.6 mg/kg, 4 days	Intellectual disability, hearing impairment, epilepsy
8	3–4 months	*S. pneumoniae*	10,490	15.7	29.2	2,096	165	26	CTX, MEPM	0.6 mg/kg, 2 days	No sequelae
9	0–1 month	*E. coli*	11,800	0.52	7.0	> 10,000	140	60	ABPC, CPR	0.6 mg/kg, 1 day	No sequelae
10	0–1 month	*E. coli*	20,360	0.2	6.9	1,913	77	50	ABPC, CTX	None	No sequelae

*H. influenzae *Haemophilus influenzae, *S. pneumoniae* Streptococcus pneumoniae, *E. coli* Escherichia coli, *WBC* White blood cell, *CRP* C-reactive protein, *N/A* Not available, *CTX* Cefotaxime, *MEPM* Meropenem, *CTRX* Ceftriaxone, *PAPM/BP* Panipenem/betamipron, *CPR* Cefpirome. *ABPC* Ampicillin

##### Objective

To evaluate longitudinal changes in HMGB1, cytokines, fever, and CRP levels, and to investigate their relationship with treatment and outcomes.

Inclusion criteria:

1. Availability of a sample collected on day 1–2.
2. At least two additional samples collected within three predefined periods (day 3–6, day 7–10, and day 11–16).

*Exclusion criteria:*

1. Lack of sufficient serial samples meeting the above criteria.

##### Cohort description

From the 26 patients in the Analysis Cohort Group, 16 were excluded due to insufficient serial samples. The remaining 10 patients (median age 0.9 years [IQR: 0.4–1.1], range 0.2–3.2 years) were included in the Longitudinal Cohort Group.

The medical records were retrospectively reviewed to investigate the clinical and laboratory findings, treatment, and prognosis (Table 2), and serial changes in fever and C-reactive protein (CRP) up to day 16 (Supplementary Fig. 1). The day of onset of fever was considered as the first day of illness, as it was the most consistently documented symptom in retrospective medical record reviews and closely aligned with the initiation of treatment. Samples were stored at −80˚C until assay.

#### AM Group

Thirteen CSF samples were obtained on the day of admission from 13 children with AM (6 males and 7 females, 3.4 years as median age [IQR 0.5–4.4], range 0.1–7.0 years) (Table 1). The causative agents were *enterovirus* (*n* = 3), *mumps virus* (*n* = 1), *Mycoplasma pneumoniae* (n = 1), and unknown (n = 8). They all had fully recovered.

#### Control group

The control subjects were 12 afebrile and noninfectious children (6 males and 6 females, 1.4 years as median age [IQR 1.7–4.6], range, 0.1–5.1 years) with neuromuscular or psychological disorders, such as epilepsy, global developmental delay, and so on. CSF samples were obtained from them as routine analysis to diagnose their diseases and they all had normal CSF cell counts, protein, and glucose.

### Determination of CSF HMGB1 and cytokine concentrations

The CSF concentrations of HMGB1 were measured with HMGB1 ELISA Kit II (Shino-test corporation, Tokyo, Japan). An anti-HMGB1 monoclonal coating antibody was adsorbed onto polystyrene microwells. HMGB1 present in the samples or the standard bound to the adsorbed antibodies, and the HMGB1/antibody complex was detected with an alkaline phosphatase-conjugated secondary antibody. The amount of captured HMGB1 was measured by determining the color produced by reagents using iMark Microplate Absorbance Reader (BIO RAD, Hercules, CA, USA). The limit of detection (LOD) was 1.0 ng/mL.

The CSF concentrations of interleukin (IL)−2, IL-4, IL-6, IL-10, tumor necrosis factor (TNF), and interferon-gamma (IFN-γ) were measured with BD Cytometric Bead Array Human Th1/Th2 Cytokine Kit II (BD Biosciences, San Jose, CA, USA). The LODs of IL-2, IL-4, IL-6, IL-10, TNF, and IFN-γ were 2.6, 2.6, 3.0, 2.8, 2.8, and 7.1 pg/mL, respectively.

### Statistical analysis

Differences between two groups for continuous variables were compared using Mann–Whitney *U* test. Three or more group comparisons were performed using Kruskal–Wallis test followed by Bonferroni-corrected Mann–Whitney *U* test. Categorical variables were compared using a Chi square test. Correlations among parameters were calculated applying Spearman correlation coefficient method. To identify differences associated with time course (day 1–2, 3–6, 7–10, and 11–16), individual (ten patients with BM), and parameters (HMGB1 and IL-6), we applied three-way ANOVA. In preparation for the analysis, values of HMGB1 and IL-6 were logarithmically transformed to correct their skewness, and then they were standardized by use of mean and SD calculated under the transformed scale to make them in uniform scale. If there was no sample in one of the four periods, it was supplemented with the mean value of the samples in the other three periods: i.e., the mean imputation method [18]. *P*-values less than 0.05 were taken to be significant. Statistical analyses were performed using StatFlex version 6.0 (Artech Co., Osaka, Japan) and Prism version 8.0 (GraphPad Software Inc., La Jolla, CA, USA).

## Results

### CSF HMGB1 concentrations in BM, AM, and controls

**Fig. 2:**
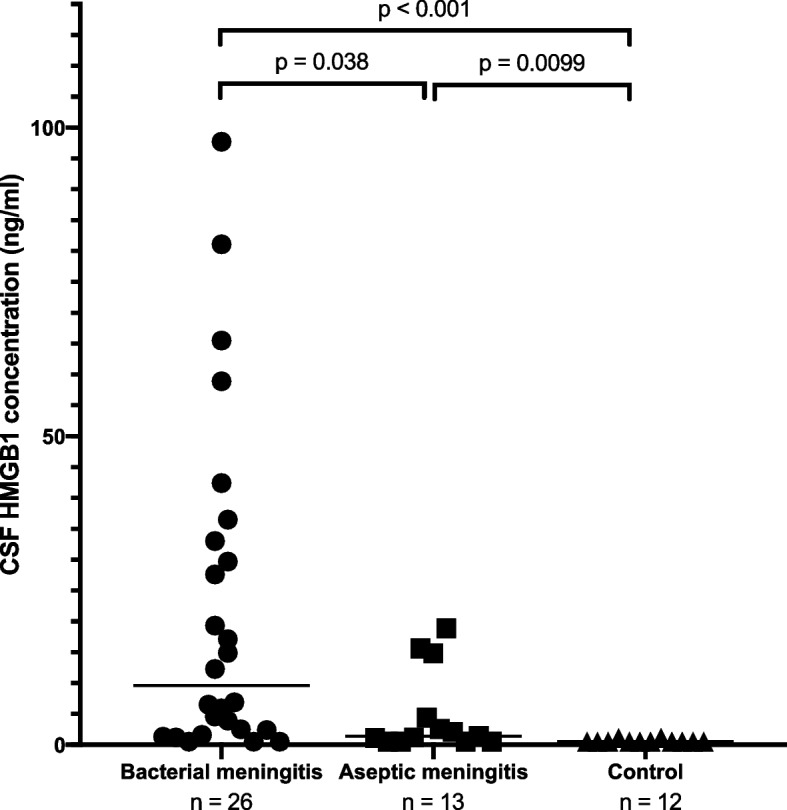
The cerebrospinal fluid (CSF) high mobility group box 1 (HMGB1) concentrations of patients with bacterial meningitis in the baseline cohort group, aseptic meningitis, and control subjects. All samples were obtained on admission. Horizontal lines indicate median values. Statistics of multiple comparisons were conducted using Bonferroni-corrected Mann–Whitney *U* test

The comparison of HMGB1 concentrations among three groups in Table 1 was shown in Fig. 2. There was no significant difference in sex among the three groups, but the ages in the AM group were significantly higher than those in the BM group. The data of BM and AM patients were taken by the first procedures of lumbar puncture, which were mainly on admission. The HMGB1 levels in the BM group ranged from < LOD to 97.7 ng/mL, with a median of 9.6 ng/mL (IQR: 2.4–32.2), and were significantly higher than those in the AM group (< LOD to 18.9 ng/mL, median 1.4 ng/mL, IQR: < LOD–4.4) and the control group (< LOD to 1.1 ng/mL, median < LOD, IQR: < LOD– < LOD) (*p* = 0.038 and *p* < 0.001, respectively). The HMGB1 levels of AM were significantly higher than those of controls (*p* = 0.0099). There were no significant differences in HMGB1 levels in the first sample between patients with and without neurological sequelae in the BM group (Supplementary Fig. 2). Similarly, no significant differences were observed when comparing HMGB1 levels among BM cases caused by *H. influenzae*, *S. pneumoniae*, *E. coli*, and other pathogens (Supplementary Fig. 2). The patients with BM caused by *S. pneumoniae* and GBS had neurological sequelae (4 and 1 cases, respectively), and the one by *P. mirabilis* died*.*

### CSF cytokine concentrations

**Fig. 3:**
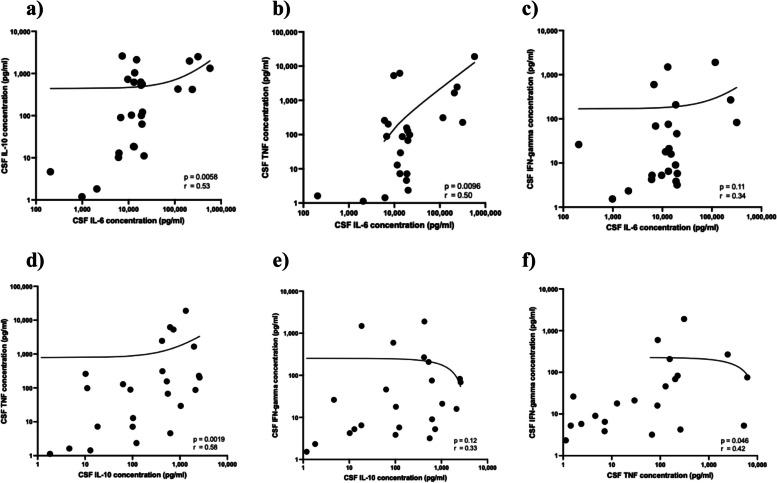
The correlations between inflammatory cytokines in 26 patients with BM in the baseline cohort group. **a** interleukin (IL)−6 and IL-10, **b** IL-6 and tumor necrosis factor (TNF), **c** IL-6 and interferon (IFN)-γ, **d** IL-10 and TNF, **e** IL-10 and IFN-γ, **f** TNF and IFN-γ. All samples were obtained on admission. The correlation line represents the linear regression fit. Statistics were conducted using Spearman correlation coefficient method

The CSF concentrations of IL-6, IL-10, TNF, IL-2, IL-4, and IFN-γ in patients with BM were 14,255.9 pg/mL as median (IQR: 7,894.7–19,978.8; range: 205.0–577,037.0), 269.9 (IQR: 18.3–704.0; range: < LOD–2,600.7), 87.9 (IQR: 5.2–251.7; range: < LOD–18,964.0), < LOD (IQR: < LOD– < LOD; range: < LOD–201.5), < LOD (IQR: < LOD– < LOD; range: < LOD–145.4), and 17.9 (IQR: < LOD–73.6; range: < LOD–1,899.8), respectively (Supplementary Table 1). CSF IL-2, IL-4, and FN-γ levels in 70%, 78%, and 39% of patients with BM were under LOD, respectively. There were significant correlations between IL-6 and IL-10, between IL-6 and TNF, between IL-10 and TNF, and between TNF and IFN-γ (r = 0.53, 0.50, 0.58, 0.42; *p* = 0.0058, 0.0096, 0.0019, 0.046, respectively) (Fig. 3). There were no significant correlations between HMGB1 and cytokines (IL-6, IL-10, TNF, and IFN-γ) (Supplementary Fig. 3). There were no significant differences in cytokine levels on first sample between with and without neurological sequelae (Supplementary Fig. 2).

### Serial CSF HMGB1 and IL-6 concentrations

**Fig. 4:**
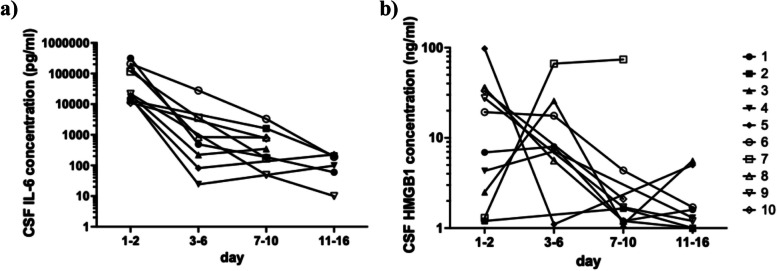
The serial IL-6 **(a)** and HMGB1 **(b)** concentrations in 10 representative patients of BM in the longitudinal cohort group from day 1 to 16. The first day means the one on admission. Days were divided into 4 periods of 1–2, 3–6, 7–10, and 11–16. The condition that 10 patients were selected from those with BM is an existence of both the first samples on day 1 or 2 and at least 3 samples until day 16. The samples derived from the same patients were connected by straight lines. Differences in time course, individual, and parameter were assessed by three-way ANOVA

A total of 30 CSF samples were obtained from 10 patients in Table 2 as a routine examination on the 1st to 16th day of diseases. Numbers of sampling from 10 patients during 1–2, 3–6, 7–10, and 11–16 were 10, 8, 6, and 6, respectively (Fig. 1). The serial concentrations of HMGB1 and IL-6 were shown in Fig. 4. The peak concentrations of IL-6 were observed at day 1–2 in all patients, which once dropped, and then second peaks were observed in 5 patients. On the other hand, the peak concentrations of HMGB1 were after day 3 in 5 patients. HMGB1 levels in Patient 7 showed a continuous upward trend across the three sampling points, peaking in the final period. A three-way ANOVA revealed significant effects for time course (*p* < 0.001), between-individual differences (p = 0.0058), and the interaction between time course and parameters (*p* = 0.018), indicating that the patterns of time-dependent changes in HMGB1 and IL-6 were significantly different across patients. The patient 7 had neurological sequelae of intellectual disability, hearing impairment, and epilepsy, and the patient 6 had hearing impairment. The other patients had no neurological sequelae. The clinical data of sequential transitions of fever and CRP were obtained from these patients (Supplementary Fig. 1). Six patients (60%) had fever again once after they fell during day 3–5, and they all received dexamethasone treatment. The rise of CRP for the second time was observed in 6 patients (60%), and the days in which CRP began to go up again were from 5 to 10.

## Discussion

Previously, HMGB1 levels in human CSF have been reported in several diseases, including meningitis [19, 20], encephalopathy [21], traumatic brain injury [22], subarachnoid hemorrhage [23], and neuromyelitis optica [24, 25]. And previous reports of HMGB1 in human meningitis have been considered only for specimens with a relatively small sample size and at early time points of the disease. Here, we provide the first report of serial CSF HMGB1 levels in human BM.

Our data show that CSF HMGB1 levels were elevated in most patients with BM. However, HMGB1 levels at admission were not consistently higher in patients with neurological sequelae or fatal outcomes. Three-way ANOVA performed by using the uniform scale transformed data revealed that the pattern of time-dependent changes in IL-6 and HMGB1 levels was significantly different. Additionally, no significant correlations were observed between HMGB1 and cytokine levels, which may reflect HMGB1's role as a late-phase inflammatory mediator [11]. Unfortunately, due to the limited availability of serial CSF samples, including the fatal case, only two cases with neurological sequelae were available for the time-course analysis, preventing a full evaluation of HMGB1 dynamics in the most severely affected patients.

In the BM, antibiotics cause rapid lysis of bacteria and enhanced release of endotoxin and exotoxin into the CSF, which can stimulate astrocytes, microglia, and cerebral capillary endothelia, to produce cytokines such as TNF-α, IL-1, IL-6, and IL-8 [26]. The cytokines make attractant of leukocytes to the CSF through activation of adhesion molecules, and leukocytes release proteolytic products and toxic oxygen radicals [26]. A complex network of cytokines, chemokines, proteolytic enzymes, excitatory aminoacids and oxidants take part in the inflammatory cascade that leads to brain edema, ischemia, and neuronal injury [26]. Intracerebroventricular administration of HMGB1 increases brain TNF, IL-1, and IL-6 expression and induces fever, anorexia, and weight loss in mice [27, 28]. Thus, elevated HMGB1 concentrations in CSF of patients might be involved in inflammatory mechanisms of BM.

The fact that CSF HMGB1 levels in the majority of the patients with BM have increased for the first several days is not inconsistent with previous reports. HMGB1 is thought to act as a late mediator because the kinetics of its release is delayed compared with most other cytokines such as IL-6 in sepsis [11]. IL-6 is a cytokine to play an important role as a primary mediator in the inflammatory responses [29, 30]. In our data, inflammatory (IL-6, TNF) and anti-inflammatory (IL-10) cytokines had positive correlations among the first samples. And IL-6 decreased promptly after treatment. Although IL-6 levels are often highly elevated in patients with BM, some studies suggest that TNF-α, rather than IL-6, may be more closely associated with neurological sequelae [29]. Similarly, serum HMGB1 levels in sepsis patients have been reported to be higher in non-survivors compared to survivors [8], suggesting that HMGB1 could be associated with severe disease progression. In our study, CSF HMGB1 levels showed a delayed increase after day 7 only in Patient 7, who developed severe neurological sequelae. Although this finding is based on a single case, it may reflect HMGB1’s role as a late-phase inflammatory mediator.

The release of HMGB1 occurs in two different ways, active secretion from living inflammatory cells and passive release from necrotic cells [32, 33]. The elevated CSF HMGB1 concentrations in patients with BM may originate in both ways because the pathogenesis of BM includes strong inflammation and neuronal cell death [34]. As shown in Fig. 4a, CSF IL-6 concentrations peaked at day 1–2 in all patients, with a secondary peak in half of the patients. Similarly, fever and CRP levels also rose again in approximately half of patients (Supplementary Fig. 1), suggesting persistent inflammation. This secondary increase could result from renewed active secretion by living inflammatory cells after steroid discontinuation. However, based on the CSF HMGB1 dynamics observed during the first several days, we considered that steroid therapy alone might not be sufficient to suppress HMGB1 levels, as it primarily targets inflammatory responses and may not prevent HMGB1 release from necrotic cells. This persistent release could maintain or exacerbate inflammation even after steroid treatment. Passive immunization with anti-HMGB1 antibodies significantly protects against lethal endotoxemia in mice, even when treatment was delayed 2 h after LPS exposure [8, 9]. The microRNA, miR-141-3p, suppressed inflammatory response through the downregulation of HMGB1 in astrocytes of BM rat model [35]. These reports suggested that HMGB1 was expected to be potential therapeutic targets [15, 16]. There might be a room for improve neurological outcome to use an adjunctive anti-HMGB1 therapy.

The present study has several limitations. First, the sample size was too small to prove the correlation between delayed elevation of HMGB1 and poor prognosis statistically. Due to the success of vaccination programs, the number of BM cases caused by vaccine-preventable pathogens has decreased, limiting the size of our study population. Second, we could not obtain consecutive samples from all the BM patients because of the retrospective study, ethical issues, and a various clinical need for collection of CSF samples. A further large-scale prospective study is necessary to clarify them. Third, the control group included patients with epilepsy and other conditions due to the limited availability of CSF samples from healthy children, which may affect the consistency of the control data. However, most HMGB1 levels in the control group were below the detection limit, minimizing potential bias. Finally, this study does not directly prove a causal relationship of elevated HMGB1 for neurological sequelae in human, not just the result of tissue damage. This problem can be indirectly complemented by the in vivo experiments mentioned above.

In conclusion, CSF HMGB1 levels are elevated in most patients with BM. Although delayed HMGB1 elevation was observed in only one patient in our study, this finding suggests a potential association with poor prognosis in BM, warranting further investigation in larger cohorts.

## Supplementary Information

Supplementary Material 1.

Supplementary Material 2.

## Data Availability

The datasets used and/or analyzed during the current study are available from the corresponding author on reasonable request.

## Abbreviations

CSF: Cerebrospinal fluid
HMGB1: High mobility group box 1
BM: Bacterial meningitis
AM: Aseptic meningitis
IL: Interleukin
CRP: C-reactive protein
TNF: Tumor necrosis factor
IFN-γ: Interferon-gamma
LOD: Limit of detection
GBS: Group B *Streptococcus*
