# Supplementary figures and images for: Serial cerebrospinal fluid concentrations of high mobility group box 1 in bacterial meningitis: a retrospective cohort study
Source: BMC Infect Dis. 2025 Jan 23;25:107. doi: 10.1186/s12879-025-10476-7 (PMC11756128; doi:10.1186/s12879-025-10476-7)

## Supplementary figure 2

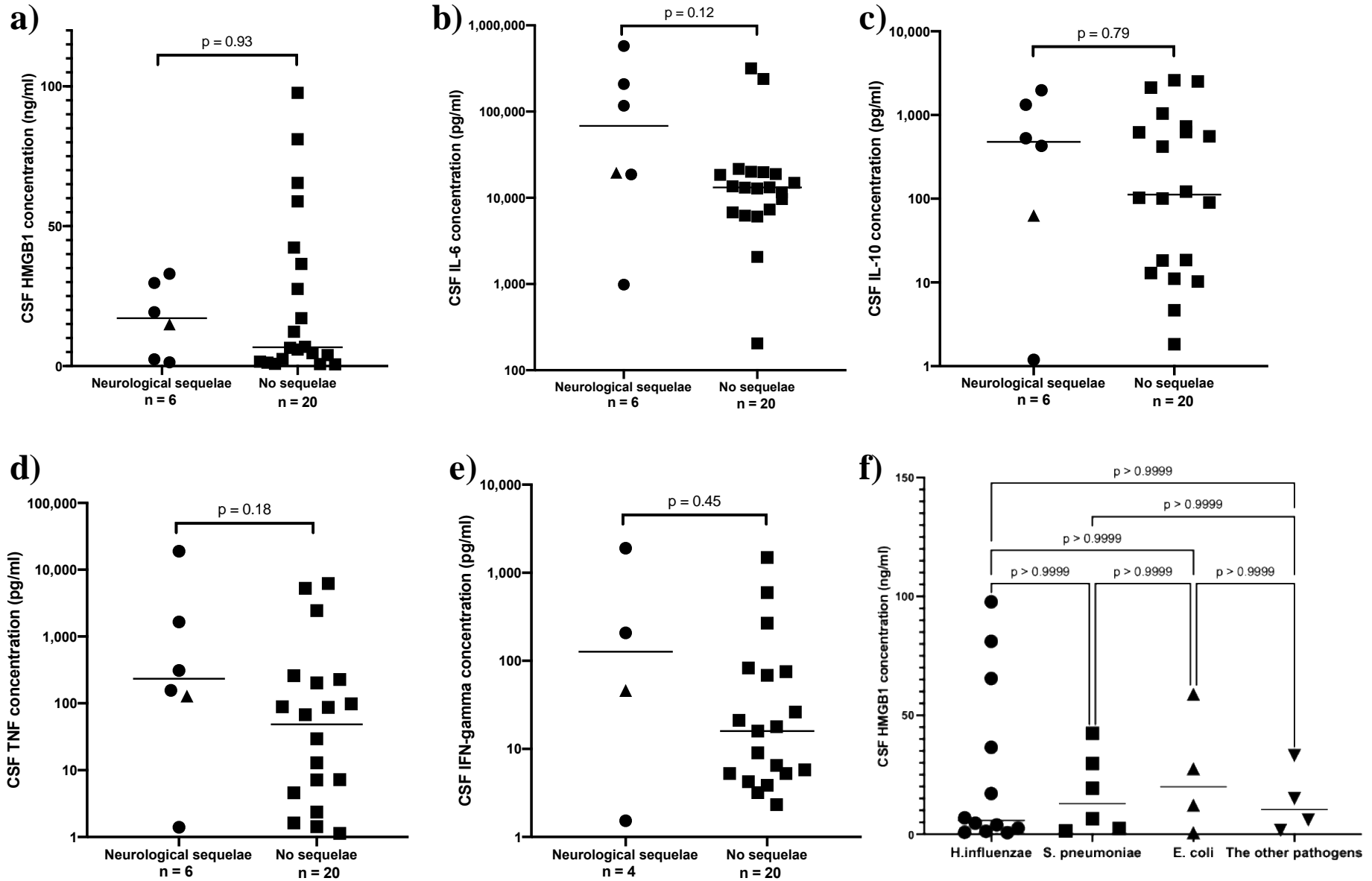

## Supplementary figure 3

a)

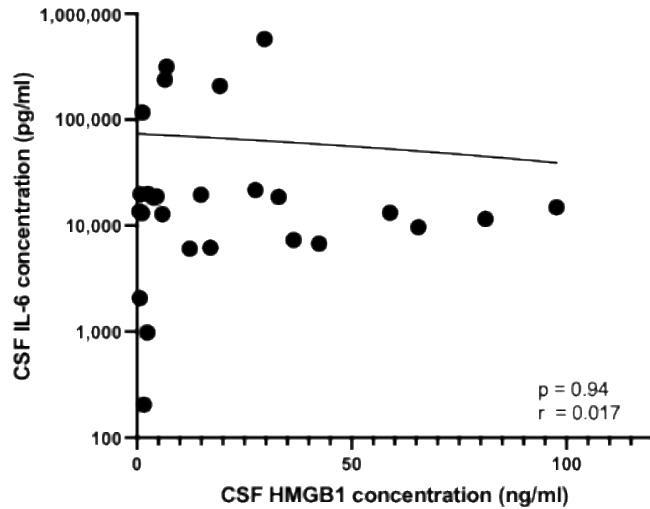

b)

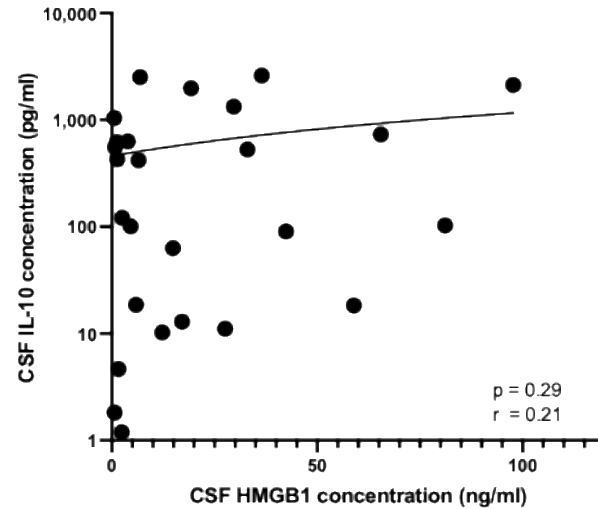

c)

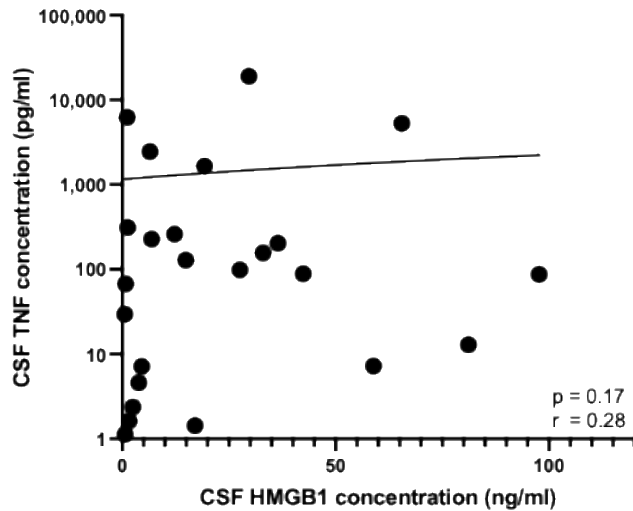

d)

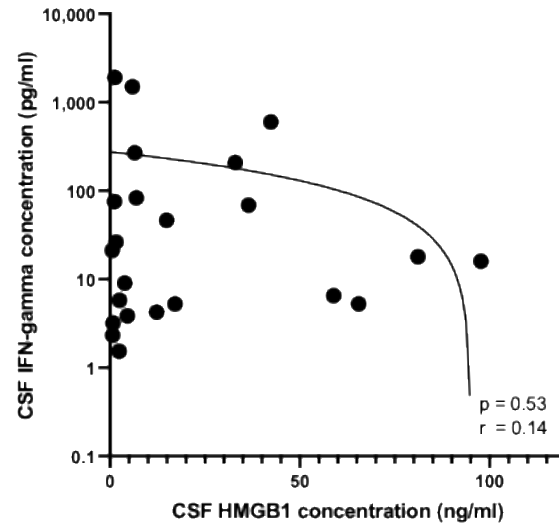

Supplement: Supplementary file 1 — Supplementary Material 1. [file 12879_2025_10476_MOESM1_ESM.pdf]
