# Supplementary material for: Serial cerebrospinal fluid concentrations of high mobility group box 1 in bacterial meningitis: a retrospective cohort study
Source: BMC Infect Dis. 2025 Jan 23;25:107. doi: 10.1186/s12879-025-10476-7 (PMC11756128; doi:10.1186/s12879-025-10476-7)
Supplement: Supplementary file 2 — Supplementary Material 2. [file 12879_2025_10476_MOESM2_ESM.docx]

| **Supplementary Table 1.** Cerebrospinal fluid cytokine concentrations in patients with bacterial meningitis. | | | | | | |
| --- | --- | --- | --- | --- | --- | --- |
| Sample ID | IL-6  (pg/mL) | IL-10  (pg/mL) | TNF  (pg/mL) | IFN-γ  (pg/mL) | IL-2  (pg/mL) | IL-4  (pg/mL) |
| 1 | 983.1 | <LOD | <LOD | <LOD | <LOD | <LOD |
| 2 | 208185.0 | 1978.3 | 1656.4 | <LOD | <LOD | <LOD |
| 3 | 577037.0 | 1329.2 | 18964.0 | <LOD | <LOD | <LOD |
| 4 | 6767.6 | 90.2 | 88.8 | 596.3 | 21.1 | <LOD |
| 5 | 7308.3 | 2600.7 | 202.7 | 68.6 | <LOD | <LOD |
| 6 | 6201.7 | 12.9 | <LOD | <LOD | <LOD | <LOD |
| 7 | 18435.4 | 626.3 | 4.6 | 9.0 | <LOD | <LOD |
| 8 | 11573.5 | 102.7 | 12.9 | 17.9 | 3.1 | 2.7 |
| 9 | 18878.7 | 100.8 | 7.1 | <LOD | <LOD | <LOD |
| 10 | 316704.2 | 2514.0 | 227.2 | 82.8 | <LOD | 145.4 |
| 11 | 9653.7 | 729.9 | 5291.5 | <LOD | <LOD | <LOD |
| 12 | 13594.1 | 1040.9 | 29.4 | 21.2 | 3.1 | 3.0 |
| 13 | 21611.0 | 11.1 | 98.4 | <LOD | <LOD | <LOD |
| 14 | 2066.3 | <LOD | <LOD | <LOD | <LOD | <LOD |
| 15 | 6068.3 | 10.2 | 259.9 | <LOD | <LOD | <LOD |
| 16 | 18655.6 | 527.8 | 156.3 | 207.8 | 2.8 | <LOD |
| 17 | 12831.7 | 18.5 | <LOD | 1492.0 | 4.6 | 3.7 |
| 18 | 19565.4 | 63.1 | 128.5 | 46.1 | <LOD | <LOD |
| 19 | 205.0 | 4.7 | <LOD | 26.2 | <LOD | <LOD |
| 20 | 13131.0 | 620.5 | 6202.6 | 75.3 | <LOD | <LOD |
| 21 | 20038.3 | 121.3 | <LOD | <LOD | <LOD | <LOD |
| 22 | 116914.4 | 427.3 | 310.1 | 1899.8 | 201.5 | <LOD |
| 23 | 238866.9 | 418.4 | 2449.4 | 268.2 | 106.9 | 142.8 |
| 24 | 19800.3 | 555.3 | 67.4 | <LOD | <LOD | <LOD |
| 25 | 14917.7 | 2122.9 | 87.1 | 15.9 | <LOD | <LOD |
| 26 | 13245.3 | 18.3 | 7.2 | <LOD | <LOD | 2.6 |
| LOD, limit of detection | | | | | | |
